# 立体定向放疗与手术治疗早期NSCLC临床疗效*meta*分析

**DOI:** 10.3779/j.issn.1009-3419.2020.101.50

**Published:** 2020-12-20

**Authors:** 强彬 武, 万朋 高, 家旺 朱, 强 王, 伟 张

**Affiliations:** 1 300150 天津，天津中医药大学第二附属医院急诊内科 Department of Emergency Medicine, Second Affiliated Hospital of Tianjin University of Traditional Chinese Medicine, Tianjin 300150, China; 2 300150 天津，天津中医药大学第二附属医院呼吸科 Department of Respiration Medicine, Second Affiliated Hospital of Tianjin University of Traditional Chinese Medicine, Tianjin 300150, China; 3 300121 天津，天津市人民医院胸外科 Department of Thoracic Surgery, Tianjin People's Hospital, Tianjin 300121, China

**Keywords:** 立体定向放疗, 手术, 肺肿瘤, *Meta*分析, Stereotactic body radiotherapy, Surgery, Lung neoplasms, *Meta* analysis

## Abstract

**背景与目的:**

采用循证医学方法探讨立体定向放疗（stereotactic body radiotherapy, SBRT）与手术治疗早期非小细胞肺癌（non-small cell lung cancer, NSCLC）的临床疗效有无差异。

**方法:**

检索PubMed、EMBASE、中国知网、万方等数据库中2020年6月以前发表的相关文献，由两名研究人员独立进行检索及提取数据，采用Stata 13.0软件对纳入文献中两种治疗方法的总生存率和癌症特异性生存率进行*meta*分析，根据倾向性评分匹配后研究和手术类型（肺叶切除术、肺段切除术及胸腔镜辅助手术）进行亚组分析。

**结果:**

最终纳入文献14篇，其中SBRT组15, 841例，手术组17, 708例，9篇采用了倾向性评分匹配方法。13篇为回顾性队列研究，1篇为随机临床对照试验。Meta分析结果显示手术组和SBRT组的总生存率差异有统计学意义，SBRT组的总生存率（HR=1.51, 95%CI: 1.31-1.74）劣于手术组。在手术类型的亚组分析中，SBRT组与各手术类型均无统计学差异。采用了倾向性评分匹配后，SBRT组与手术组的总生存率差异仍有统计学意义（HR=1.66, 95%CI: 1.45-1.90）。手术组和SBRT组的癌症特异性生存率无统计学差异（HR=1.12, 95%CI: 0.83-1.52）。

**结论:**

手术治疗的总生存率优于SBRT治疗，但在癌症特异性生存率上无明显优势。

## Abstract (en)

**Background and objective:**

To compare the clinical efficacy of stereotactic body radiotherapy (SBRT) versus surgery for early-stage non-small cell lung cancer through evidence based medicine analysis.

**Methods:**

A systematic search was performed in the PubMed, EMBASE, CNKI and Wan Fang databases to find studies published before June 2020. Two authors independently extracted the data and assessed the eligibility. All of the statistical analyses were performed using Stata 13.0. Subgroup analysis were performed according to the score matching study and operation type (lobectomy, segmentectomy and thoracoscopic assisted surgery).

**Results:**

Finally, 14 articles were included, including 15, 841 cases in SBRT group and 17, 708 cases in operation group. 10 articles used propensity score matching methods for survival analysis. Thirteen were retrospective cohort studies and one was randomized controlled trial. The results of *meta*-analysis showed that the overall survival rate of the surgery group and the SBRT group was statistically significant. The overall survival rate of the SBRT group (HR=1.51, 95%CI: 1.31-1.74) was inferior to that of the surgery group. In the subgroup analysis of the surgical type, there was no statistical difference between the SBRT group and each surgical type. The difference of overall survival rate between SBRT group and surgery group was statistically significant (HR=1.66, 95%CI: 1.45-1.90) in studies of propensity score matching. There was no statistically significant difference in cancer-specific survival between the surgery and SBRT groups (HR=1.12, 95%CI: 0.83-1.52).

**Conclusion:**

The overall survival rate of surgical treatment is better than that of SBRT, but it has no obvious advantages in cancer specific survival rate.

肺癌目前是临床上发病率最高的恶性肿瘤，同时也是所有肿瘤死亡原因的第一位^[1, 2]^。肺癌分为小细胞肺癌（small cell lung cancer, SCLC）和非小细胞肺癌（non-small cell lung cancer, NSCLC），其中NSCLC约占所有肺癌患者的80%。NSCLC早期患者大多需要进行包括肺叶和肺段切除的肺癌根治手术，术后根据患者病理分期决定是否需要进行辅助放化疗及综合治疗^[3, 4]^。无淋巴结转移的早期NSCLC患者接受根治性治疗后预后较好^[5]^，但肺癌患者年龄较大，且长期吸烟患者比例较高，肺功能不全等基础疾病发生率高。因此，部分早期患者，尤其是高龄患者，全身情况并不允许接受手术治疗，而此类患者大多接受放射治疗。

立体定向放疗（stereotactic body radiotherapy, SBRT），也被称为立体定向消融放射治疗（stereotactic ablation radiotherapy, SABR），通过多个精确瞄准的放射治疗束，将很高的辐射剂量传递到有限的体积，达到杀伤肿瘤细胞的目的。SBRT技术具有无创、高精确度、呼吸门控技减少呼吸对治疗的影响、多个照射野进行三维立体照射提高靶区照射剂量的同时降低对周围正常组织的损伤以及照射时间段等特点^[6, 7]^。对于早期NSCLC患者，SBRT是否可以代替或等效于传统的外科手术治疗一直存在争议。不同的临床研究结果并不完全一致。因此，在本研究中，我们对近年来SBRT与手术治疗早期NSCLC的临床研究进行了系统性分析，从总生存、癌症特异性生存等方面比较了两种治疗方法的优劣。

## 1 资料与方法

### 1.1 检索策略

检索PubMed、EMBASE、中国知网、万方数据库中已发表的立体定向放疗法与手术治疗早期NSCLC临床疗效的相关文献，检索日期截止至2020年6月。英文检索词为（Stereotactic ablation radiotherapy）OR（Stereotactic body radiotherapy）AND（Surgery）AND（Lung cancer），采用主题词与自由词相结合的方法进行检索；中文检索词为“立体定向放疗”或“立体定向放射疗法”、“手术”和“肺癌”。同时，手工检索相关文献的参考文献。检索语言为中文或英文。

### 1.2 文献纳入及排除标准

根据研究对象、干预措施、对照及结局（Patient population, Intervention, Comparison, Outcome, PICO）原则，我们从以上四个方面对文献进行筛选。（1）文献纳入标准：①纳入对象为早期NSCLC患者（早期NSCLC本研究定义为Ia期-IIb期患者）；②干预措施为SBRT与手术治疗疗效的比较；③文章提供了风险比（hazard ratio, HR）及95%可信区间（95% confidence interval, 95%CI）。（2）排除标准: ①原始数据不完整或信息不足；②评论、综述、*meta*分析等；③重复发表的文献。

### 1.3 纳入文献的质量评价

保证文献质量是高质量*meta*分析的前提条件，为保证本研究的可靠性，对预纳入的文献采用纽卡斯尔-渥太华量表（Newcastle-Ottawa-Scale, NOS）进行质量评价。本量表共计9分，主要涉及对象选择、可比性和暴露/结局三部分。评分大于6则认为是高质量文献，可被纳入研究。

### 1.4 提取数据

由两名研究人员独立进行检索，通过阅读摘要及全文确定相关文献。从纳入文献中提取以下内容：第一作者、发表年份、国别、研究类型、随访时间、年龄、样本量、肿瘤原发灶-淋巴结-转移（tumor-node-metastasis, TNM）、结局事件以及相应的风险效应值等信息，分歧之处与第三人协商解决。研究中若存在多个不同调整因素的HR值，则提取最多调整因素调整后的HR值。

### 1.5 统计学分析

采用Stata 13.0软件对纳入文献进行统计分析。临床疗效的结局事件为总生存率和癌症特异性生存率。按照倾向性评分匹配后研究和手术类型（肺叶切除术、肺下叶切除术及胸腔镜辅助手术）进行亚组分析。结果采用合并的HR值及95%CI进行描述。异质性评估采用*Q*统计量和*I*^2^检验判断，当*P* > 0.1且*I*^2^≤50%时采用固定效应模型；反之，则认为存在异质性，采用随机效应模型。发表偏倚采用漏斗图进行检测，并进行敏感性分析来验证结果的稳定性。

## 2 结果

### 2.1 文献检索结果

通过PubMed、EMBASE、中国知网、万方数据库共检索中英文文献1, 238篇。排除重复、不相关、数据不全及综述性研究后，最终纳入14篇文献^[8-21]^。具体流程见图 1。

**图 1:**
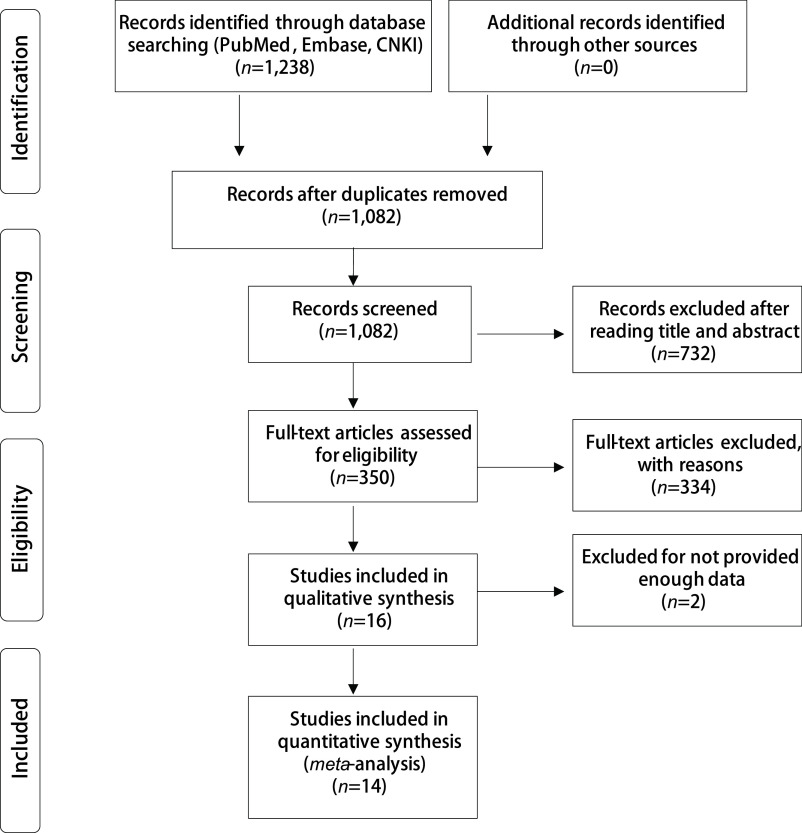
纳入文献筛选流程 The publication searching flow-chart

### 2.2 纳入文献基本特征

共纳入14篇文献，包含33, 549例患者，其中SBRT组15, 841例，手术组17, 708例。9篇采用了倾向性评分匹配方法。13篇为回顾性研究，1篇为随机对照试验。手术治疗的患者主要采用肺叶切除术，其次为肺段切除术。主要结局事件为总生存率和癌症特异性生存率。对预纳入的文献进行了质量评价，结果显示，评分均大于6分，故纳入所有文献进行分析。纳入研究的特征详见表 1。

**表 1:** 纳入研究的基本特征 The general characteristics of the included publications

Author	Year	Country	Type	Follow-up	Median follow-up (mon)			Median age (yr)			Sample size		TNM	Operation	Outcome	NOS
					SARB	Operation		SARB	Operation		SARB	Operation				
Shirvani^[8]^	2012	U.S	Retrospective	2001-2007	38	38		78.1	78.2		99	99	T1-2aN0M0	Lobectomy	OS, CSS	7
											112	112		Sublobar	OS, CSS	
Verstegen^[9]^	2013	Netherlands	Retrospective	2003-2007	30	16		70.53	67.95		64	64	T1-3N0M0	VATS-Lobectomy	OS	7
Shirvani^[10]^	2014	U.S	Retrospective	2003-2009	-	-		-	-		251	251	T1-2aN0M0	Lobectomy	OS, CSS	
Nakagawa^[11]^	2014	Japan	Retrospective	2001-2011	45	41.2		79.8	78.3		35	183	T1-2aN0M0	All	OS	6
Kastelijn^[12]^	2015	Netherlands	Retrospective	2008-2011	31.8	41.5		71.6	66.5		53	175	T1-2N0M0	All	OS	7
Chang^[13]^	2015	U.S	Prospective	2008-2014	40.2	35.4		67.1	66.7		31	27	T1-2aN0M0	Lobectomy	OS	8
Ezer^[14]^	2015	Multiple	Retrospective	2002-2009	27	38		78	76		362	1, 881	T1-2aN0M0	Sublobar	OS, CSS	7
Rosen^[15]^	2016	U.S	Retrospective	2008-2012	32	29		75.5	74.8		1, 781	1, 781	T1-2aN0M0	Lobectomy	OS	8
Eba^[16]^	2016	Japan	Retrospective	2004-2007	-	-		79	62		21	21	T1N0M0	Lobectomy	OS	6
Paul^[17]^	2016	U.S	Retrospective	2007-2013	35	35		77.6	75.6		201	201	T1-2N0M0	VATS-Sublobar	OS, CSS	6
Scotti^[18]^	2018	Italy	Retrospective	2008-2015	23	23		76.6	67.9		93	94	T1-2bN0M0	Lobectomy	OS	7
Ackerson^[19]^	2018	U.S	Retrospective	2007-2014	65	60		74	70		70	151	T1-2bN0M0	Sublobar	OS	7
Chi^[20]^	2019	U.S	Retrospective	2004-2015	-	-		75	68		12, 632	12, 632	T1-3N0M0	Sublobar、lobar	OS	8
Detillon^[21]^	2019	Netherland	Retrospective	2010-2015	33	77		72.8	73.5		36	36	T1-2aN0M0	VATS-Lobectomy	OS	6
U.S: United States; SARB: stereotactic ablative radiotherapy; SBRT: stereotactic body radiotherapy; NOS: Newcastle-Ottawa-Scale; OS: overall survival; CSS: cancer specific survival; TNM: tumor-node -metastasis.																

### 2.3 总生存率的*meta*分析

对纳入的文献进行异质性检验，结果显示存在异质性（*P* < 0.01, *I*^2^=89.7%），故采用随机效应模型，合并HR值为1.51（95%CI: 1.31-1.74），说明手术组和SBRT组的总生存率差异有统计学意义。在手术类型的亚组分析结果中，SBRT组与肺叶切除术（HR=1.54, 95%CI: 0.99-2.40）、肺段切除术（HR=1.29，95%CI: 0.86-1.92）及胸腔镜辅助手术组（HR=1.84, 95%CI: 0.36-2.39）均无差异，见图 2。9篇研究采用了倾向性评分匹配，对其进行*meta*分析，结果存在异质性（*P* < 0.01, *I*^2^=87.2%），故采用随机效应模型。结果显示手术组和SBRT组的总生存率差异仍有统计学意义（HR=1.66, 95%CI: 1.45-1.90），见图 3。

**图 2:**
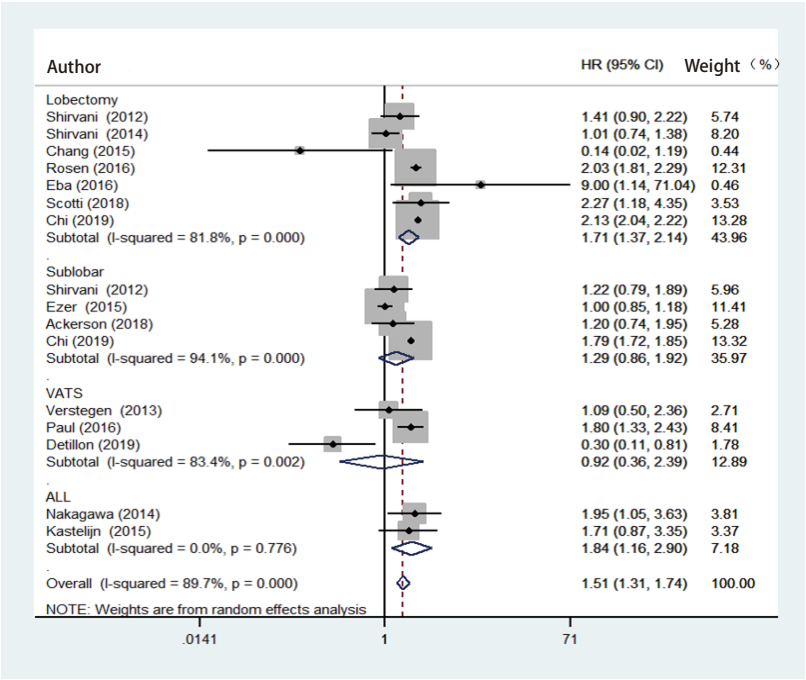
SBRT组与手术组总生存率的比较的森林图 Forest plot of the overall survival analysis between SBRT and operation

**图 3:**
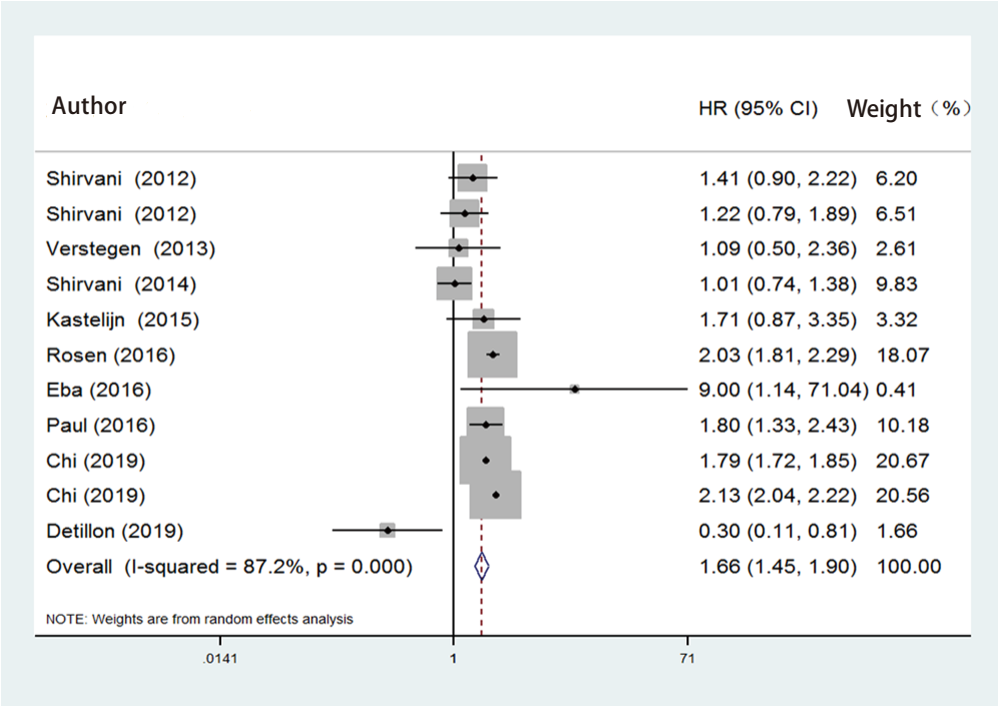
倾向性评分匹配研究中SBRT组与手术组总生存率的比较的森林图 The forest of overall survival analysis between SBRT and operation in studies of propensity score matching

### 2.4 癌症特异性生存率的*meta*分析

在随机效应模型中，手术组和SBRT组的癌症特异性生存率没有统计学差异（HR=1.12, 95%CI: 0.83-1.52）。在手术类型的亚组分析结果中，SBRT组与肺叶切除术（HR=1.00, 95%CI: 0.59-1.70）、肺段切除术（HR=1.09, 95%CI: 0.67-1.78）均无统计学差异，见图 4A。3篇研究采用了倾向性评分匹配，对其进行*meta*分析，结果显示手术组和SBRT组的癌症特异性生存率也无统计学差异（HR=0.98, 95%CI: 0.66-1.46），见图 4B。

**图 4:**
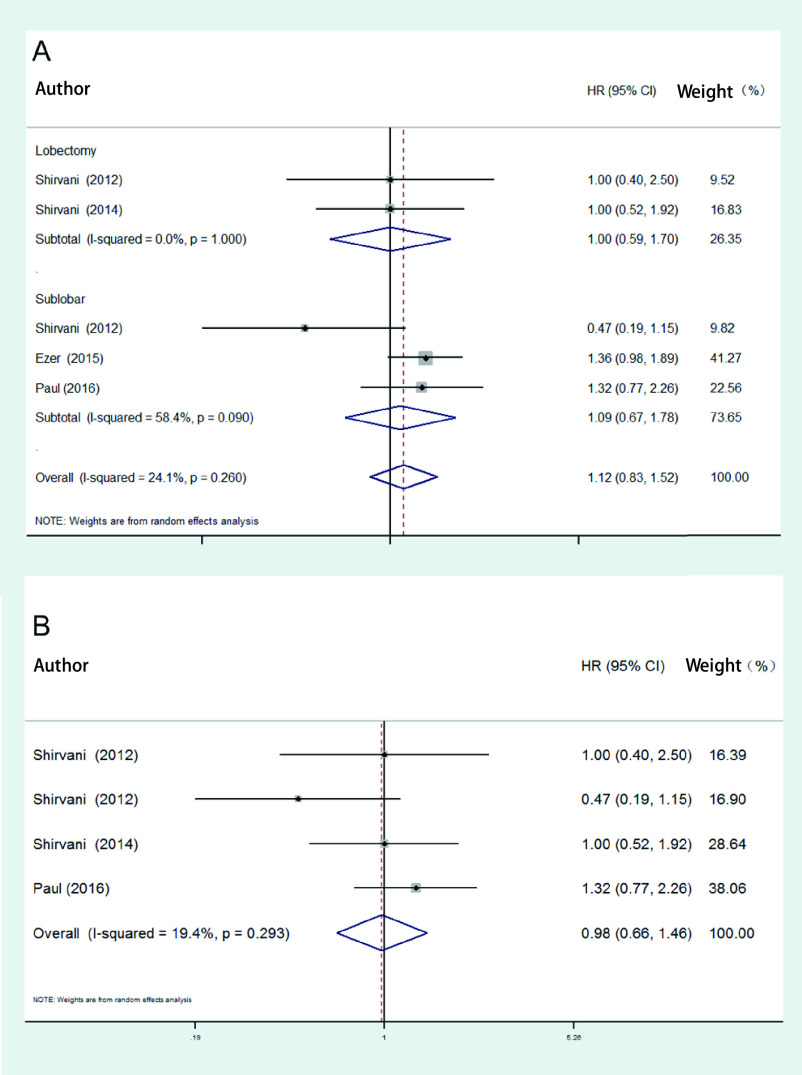
SBRT组与手术组癌症特异性生存率的比较。A：所有纳入研究；B：倾向性评分匹配研究。 The forest of cancer specific survival analysis between SBRT and operation. A: All studies included in the meta-analysis; B: Studies of propensity score matching.

### 2.5 发表偏倚及敏感性分析

采用漏斗图对纳入文献进行发表偏倚评价。对有无倾向性评分匹配的研究分别进行发表偏倚检测。图 5A、图 5B显示，SBRT组与手术组总生存率有5处散点落在可信区间外，存在发表偏倚。图 5C、图 5D显示，SBRT组与手术组癌症特异性生存率没有明显的发表偏倚。敏感性分析结果显示，纳入文献中删除任一篇文献对剩余文献合并效应值均无明显影响，证实了本研究最终结果的稳定性，见图 6。

**图 5:**
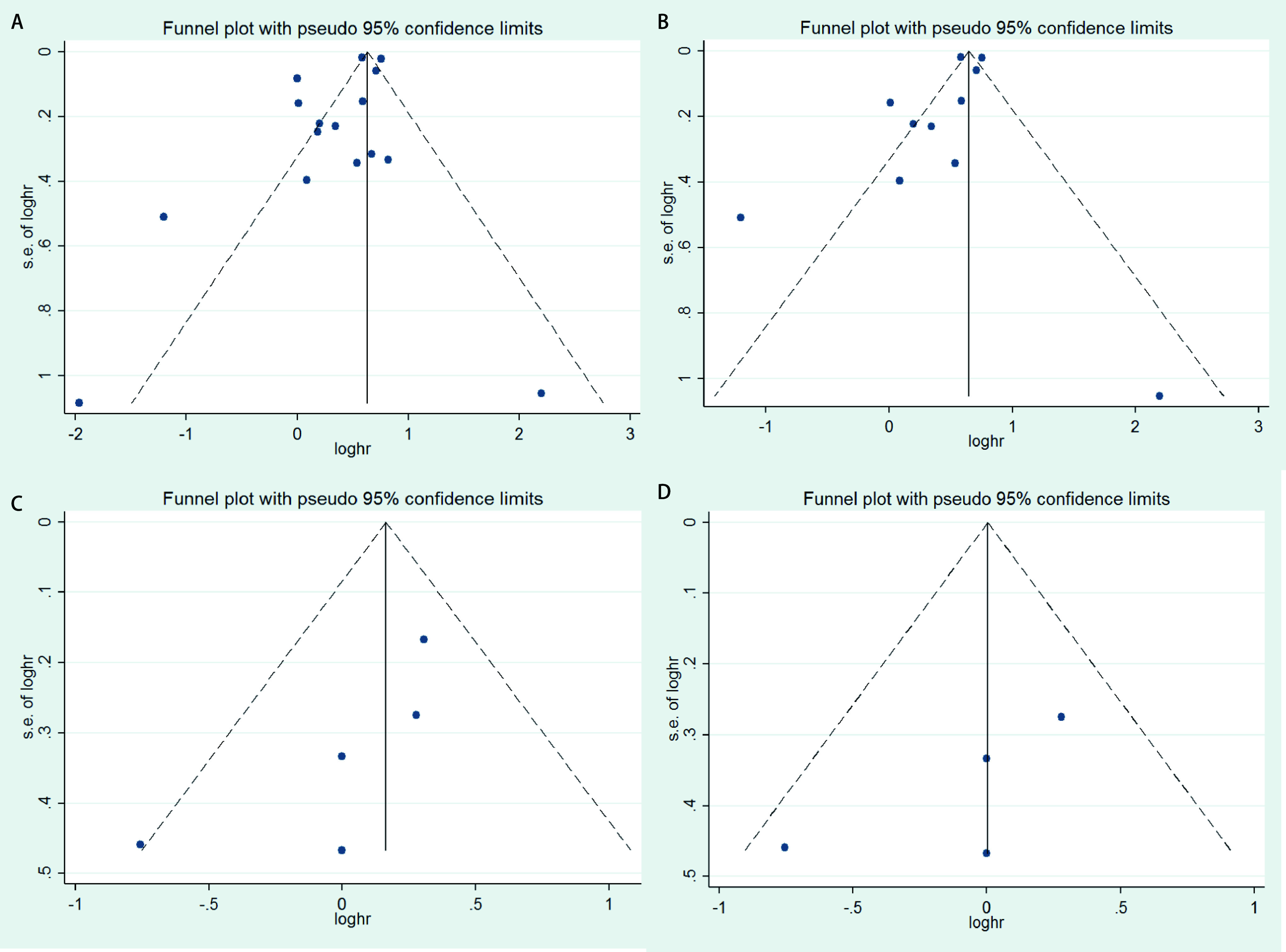
发表偏倚检测。A：总生存率研究的漏斗图；B：倾向性评分匹配后总生存率研究的漏斗图；C：癌症特异性生存率研究的漏斗图；D：倾向性评分匹配后癌症特异性生存率研究的漏斗图。 *Begger*'funnel plot of publication bias. A: Plot for overall survival; B: Plot for overall survival in studies of propensity score matching; C: Plot for cancer specific survival; D: Plot for cancer specific survival in studies of propensity score matching.

**图 6:**
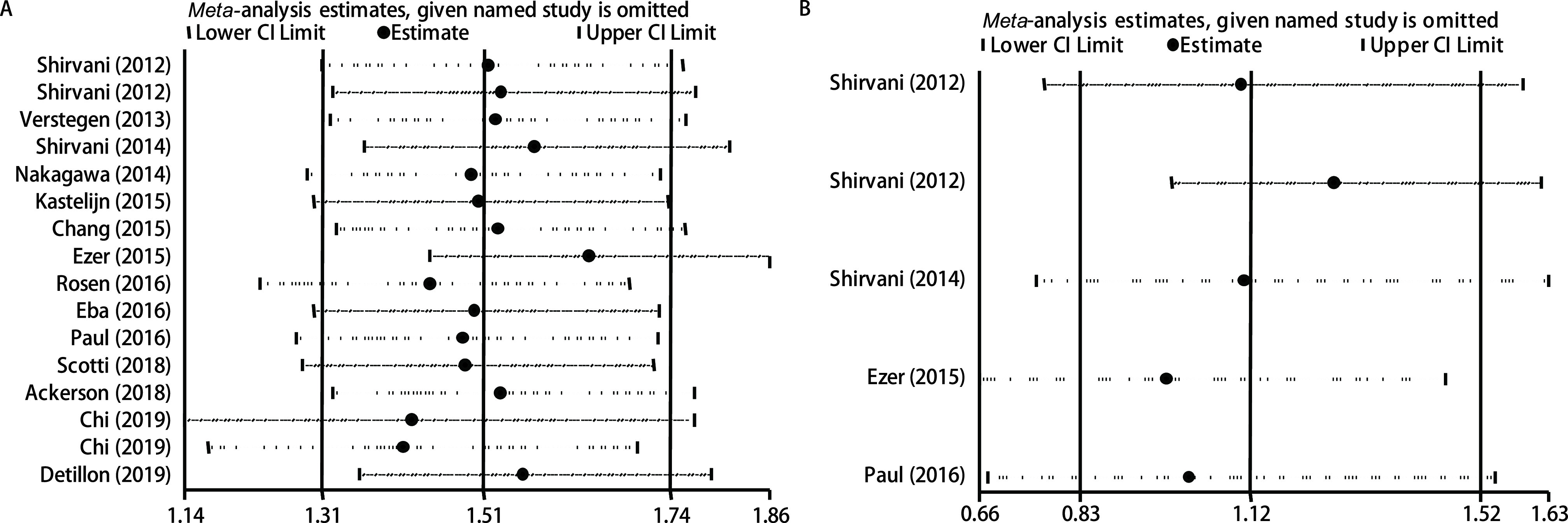
纳入研究的敏感性分析。A：总生存率研究的敏感图；B：癌症特异性生存率研究的敏感图。 Sensitivity analysis of the included studies. A: Sensitivity analysis for overall survival; B: Sensitivity analysis for cancer specific analysis.

## 3 讨论

NSCLC约占肺癌诊断患者的80%，其中约1/5的患者为早期，对于此类患者大多推荐根治性手术治疗，且术后患者远期生存率较高。然而，对于有潜在合并症的患者，特别是老年患者，由于健康状况或个人意愿等因素无法接受根治性手术治疗。此时，放疗可以被认为是一种有效的替代治疗方案。

随着放疗技术的不断改进，尤其是SBRT/SABR技术的广泛应用于临床，放疗效果得到了明显提高的同时，放疗相关并发症的发生率明显降低。已有研究表明，与肺癌根治手术相比，SBRT/SABR对于早期NSCLC患者的临床疗效并不逊色。尤其是2015年美国MD.Anderson肿瘤中心Chang等^[22]^进行的一项前瞻性临床对照研究对比SBRT *vs* 肺叶切除术治疗早期NSCLC患者的临床疗效，并发表在了*Lancet Oncology*上。研究结果显示SBRT组患者3年总生存率优于手术组，而无疾病进展生存率无统计学差异。研究结果认为SBRT可能是早期NSCLC患者的有效治疗手段，但由于样本量较小和较短的生存随访时间，期结论有待进一步相关临床研究证实。因此，美国国家综合癌症网络（National Comprehensive Cancer Network, NCCN）肺癌临床诊疗指南中将SBRT放疗推荐为除手术外治疗T1-3N0M0 NSCLC最有利的治疗选择。但也有临床研究认为，SBRT在早期NSCLC患者局部控制率和远期生存率方面不如根治性手术，建议在身体条件允许的情况下，尽量接受肺癌根治性手术治疗以最大限度地提高患者的远期生存率，改善患者预后。一项队列研究^[20]^入组了104, 709例NSCLC患者，其中42, 508例接受了手术治疗，6, 065例接受了SBRT治疗。研究结果认为接受手术和区域淋巴结清扫的NSCLC患者长期生存率明显高于接受SBRT患者。

因此，SBRT *vs* 手术治疗早期NSCLC是否存在差异仍存在一定的争议。我们采用循证医学的方法，对SBRT *vs* 手术治疗早期NSCLC的临床研究进行了系统检索，并对患者生存期数据进行了合并，进一步明确上述两种治疗方案的优劣。研究最终纳入文献14篇，13篇为回顾性队列研究，1篇为随机临床对照试验，9篇采用了倾向性评分匹配方法。其中SBRT组15, 841例，手术组17, 708例。*Meta*分析结果显示手术组和SBRT组的总生存率差异有统计学意义，SBRT组的总生存率（HR=1.51, 95%CI: 1.31-1.74）劣于手术组。在手术类型的亚组分析中，SBRT组与各手术类型均无统计学差异。我们认为，手术治疗的总生存率优于SBRT治疗，但在癌症特异性生存率上无明显优势。

因此，我们对现有研究数据进行合并分析认为早期NSCLC患者接受手术治疗的远期生存总率优于接受SBRT患者。因此，在患者身体情况允许且愿意手术治疗的情况下，应推荐接受手术治疗，以提高患者的远期生存。但对于无法接受或不愿接受手术的早期NSCLC患者，SBRT可能是一个合理的选择。
